# Effectiveness of a Uniquely Designed Oral Appliance on Obstructive Sleep Apnea Control: A Pilot Study

**DOI:** 10.1055/s-0041-1735933

**Published:** 2022-02-18

**Authors:** Denise Fernandes Barbosa, Miguel Meira e Cruz, Marcelo Corrêa Alves, Edilson Zancanella, Fausto Berzin, Almiro José Machado Júnior

**Affiliations:** 1 Division of Surgical Sciences, Department of Otorhinolaryngology, School of Medical Sciences, University of Campinas, UNICAMP, São Paulo, Brazil; 2 Sleep Unit, Centro Cardiovascular da Universidade de Lisboa, Lisbon School of Medicine, Lisbon, Portugal; 3 Research Laboratory on the Neuroimmune Interface of Pain São Leopoldo Mandic College, Campinas, São Paulo, Brazil; 4 ESALQ, University of Sao Paolo, Piracicaba, São Paulo, Brazil; 5 Department of Odontology, FOP – UNICAMP, Piracicaba, São Paulo, Brazil

**Keywords:** obstructive sleep apnea syndrome, oral appliance therapy, upper airway

## Abstract

**Objectives** Obstructive sleep apnea is an inflammatory, chronic, and evolutive disease often needing adequate treatment and follow-up. The oral appliance (OA) is an accepted alternative therapy for obstructive sleep apnea (OSA) control. Due to greater adherence, OA with mandibular advancement (OA _m_ ) is being recommended treatment for patients who refuse or do not tolerate continuous positive airway pressure. The mode of action of OA _m_ is to promote the advancement of the mandible or tongue with a subsequent increase in the tone of the pharyngeal muscles and the permeability of the upper airway, but most OA _m_ use conventional models as reference, analogic, or digital, dissociating dental arches of the skull structures.

**Materials and Methods** A retrospective longitudinal study of 33 OSA patient treated with a different OA _m_ , that use Camper plane as reference with skull structures for dental arches disocclusion, where polysomnographic, cephalometric measures, and subjective data from questionnaires pre- and post-treatment were assessed and correlated. Descriptive analysis, correlated Chi-square tests, and basic statistics were used. Generalized linear mixed model for repeated measure and post hoc Tukey–Kramer test compares the variables pre- and post-treatment. Shapiro–Wilk test and Pearson's correlation coefficients were used. All statistical tests were set in 5% level of significance.

**Results** Regarding polysomnography data, there was a significant association between apnea hypopnea index (AHI) with oxygen saturation, arousal index (AI) and the maximum heartbeats, and sleep improvement and health risk reduction. Additionally, from cephalometric data, it was found a significant association between the tongue posture with the soft palate, hioyd-C3 and, lower and posterior airway. When both parameters are correlated, there are a significant dependent association with hyoid bone position with AHI and AI. The limitation of this study was the two-dimensional image used without provide volumetric measurements, but this limitation was reduced with the follow-up polysomnography parameters.

**Conclusion** In this pilot study, DIORS OA _m_ as an uniquely designed device using Camper plane as a reference for disocclusion was effective in the control of OSA.

## Introduction

Obstructive sleep apnea (OSA) is a chronic, inflammatory, and progressive disease. 1 2 Its prevalence in the general population is between 9 and 38%, and varies according to age and gender. 3 The diagnosis 4 requires either signs/symptoms (e.g., associated sleepiness, fatigue, insomnia, snoring, subjective nocturnal respiratory disturbance, or observed apnea) or associated medical or psychiatric disorder (i.e., hypertension, coronary artery disease, atrial fibrillation, congestive heart failure, stroke, diabetes, cognitive dysfunction, or mood disorder) coupled with five or more predominantly obstructive respiratory events (obstructive and mixed apneas, hypopneas, or respiratory effort-related arousals, as defined by the American Academy of Sleep Medicine scoring manual) per hour of sleep during polysomnography (PSG). Alternatively, a frequency of obstructive respiratory events 15/hour satisfies the criteria, even in the absence of associated symptoms or disorders.

Clinical symptoms vary depending on the type, frequency, and intensity of the respiratory abnormality. 5 6 Normal rates of apnea should also be treated when associated with snoring, although some controversial issues persist regarding the therapeutic criteria for snoring itself. 7

The most recommended devices for usage by American Academy of Sleep Medicine and the American Academy of Dental Sleep Medicine 8 are the oral appliance with mandibular advancement (OA _m_ ) for the treatment of primary snoring, mild and moderate obstructive sleep apnea (OSA), and the continuous positive airway pressure (CPAP) to moderate to severe OSA. In addition, OA _m_ can be considered after CPAP has been failed in nonadherent patients treatment or in patient preference in therapy choosing. 9 10

The new generation of OA _m_ devices presents considerable advances in design, construction techniques, and individualization capacity. Considering the assumptions of design, construction, and individualization, OA _m_ can further impact the effectiveness of oral appliance therapy (OAT). 8

One of the problems related to respiratory disorders is the maxillomandibular relationship, both vertically, sagittal and transversal, 11 significant differences existed in the cranio-facial morphology of patients with OSA and the healthy population. In addition to this relationship, which is so important, most malocclusions are treated based on a conventional analog or digital model, 12 which does not faithfully reproduce their interrelationships with cranial structures. These therapeutic proceed can bring important repercussions in the lives of these patients for not considering the intimate interrelationship of the dental arches with the craniofacial structures. 13 Likewise, most OA _m_ are structured, using models dissociated from their relationship with craniometric structures and, consequently, with the muscles involved in mandibular protrusion movements.

The functional anatomic factors leading to oropharynx and hypopharynx airway collapse in OSA, 14 15 are in part related to the retracted position of the mandible and tongue with sagging soft palate. The principal mechanism of action of OA _m_ is by promoting the advancement of the mandible or tongue 14 16 because simple active anterior movement of the tongue or mandible can increase cross sectional airway size in subjects with and without OSA 17 18 and, increasing the pharynx muscles tone and therefore the airway patency. Ideally, these situations could be achieved in the OA _m_ mode of action simultaneously. 14 19 20 21 22

One of the first functional mandibular activator devices was developed in Europe in the early 20th century which became a universal device widely used, thanks to Viggo Andresen. The removable activator devices were built to redirect the pressure of facial muscles and masticatory onto teeth and support structures to improve dental arrangement and occlusal relationships. This author used the Camper plane for diagnosis and follow-up. The Camper plane is a plane established by superior border of the tragus left and right to the lowest point of ala border. 23

In OAT, the neuromuscular system will be activated through OA _m_ maintaining upper airway patency, toning the oropharynx muscles, thus preventing collapse between the tissues of the oropharynx and tongue base, 24 and improving tongue posture. 25 The OA _m_ cannot exceed anatomical physiological limits. Therefore, the choice of OA _m_ for the treatment of OSA should be considered by Dental Sleep Medicine. 21 24 26 27 28

In searching for functional balance, the DIORS OA _m_ design 29 considered these two situations because it was designed based on functional anatomy of buco-dental biology. The OA _m_ disocclusion is guided to Camper plane based on fixed individual skull structures. At same time, it stimulates the tongue for advancement, promoting lip sealing which further improve the airway patency.

In Dental Sleep Medicine clinical practice, 8 there are diagnostic and complementary exams with objective and subjective parameters need for records as polysomnography exam for diagnosing OSA, intra- and extraoral photographic examination to assess facial patterns, oral conditions, and follow-up cephalometric analyze of the airway space, which makes it possible to check the mandible and tongue position in relation bone structures 14 30 31 32 33 and models of the dental arches for recording and making the OA _m_ . In addition to these objective parameters, the Epworth sleepiness scale is a subjective parameter, of proven validity, used for the evaluation and monitoring of the patient after treatment. 8

Thus, the aim of this pilot study is to compare the impact of OA _m_ that use Camper plane for reference to disocclusion, on the upper airways in snore and OSA patients, in the pre- and post-treatment in a private dental office through cephalometric analysis, and the effectiveness of this different OA _m_ through polysomnographic parameters. Also, verify if there is a correlation with these variables associated with clinical practice.

## Materials and Methods

### Study Design

**Fig. 1:**
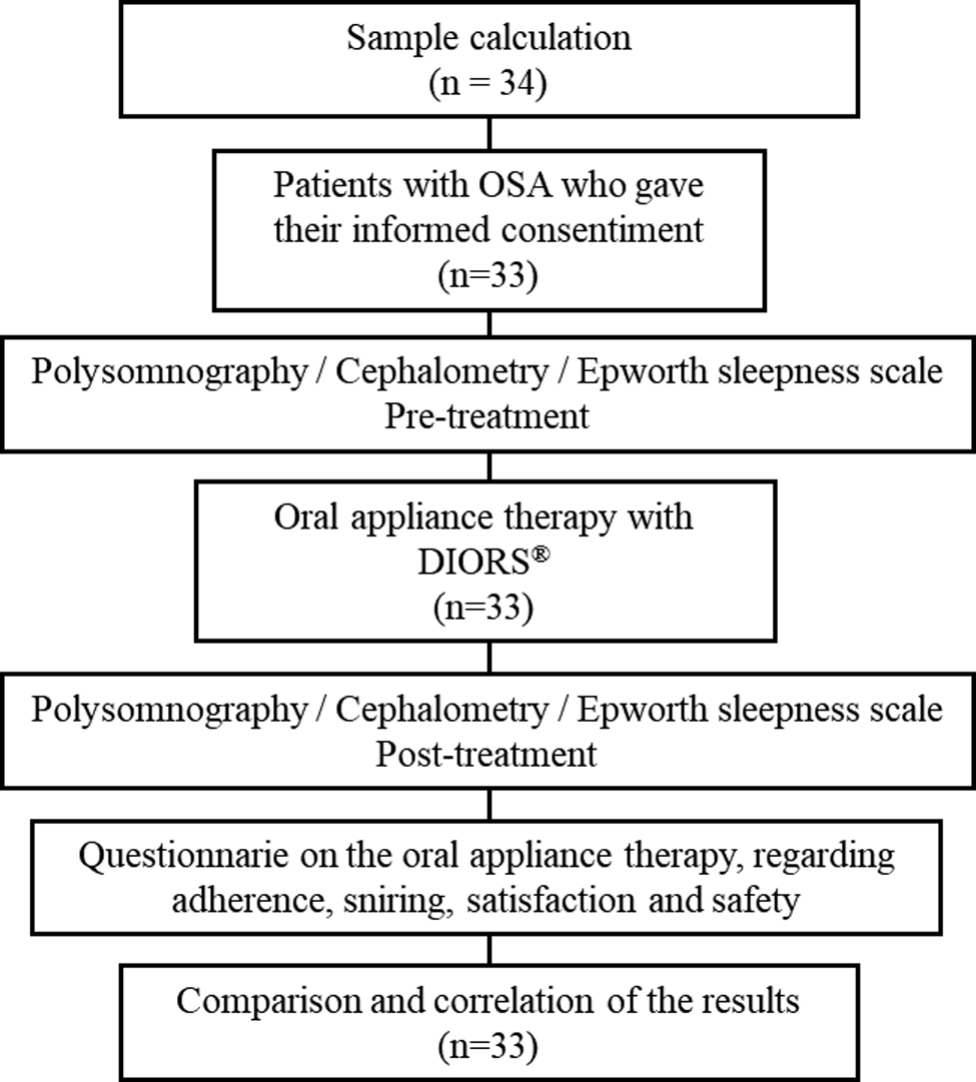
Diagram of study development.

A retrospective longitudinal study was developed from dental sleep medical records of OSA patients treated with DIORS OA _m_ , manufactured by the first author in her dental office, Jundiaí, SP, Brazil. The results were measuring and comparing by objective (polysomnography and cephalometric data) and subjective (Epworth sleepiness scale and adherence, symptoms, satisfaction, and safety questionnaires) data pre- and post-treatment. The data analysis was conducted by applying quantitative techniques and multiple comparisons ( Fig. 1 ).

The study was approved by the School of Odontology of Piracicaba UNICAMP Ethics Committee, SP, Brazil (CAAE: 20672219.3.0000.5418 P.N. /4.034.661). This study counted patients attending in private dental office with authorized and formalize participation with the informed consent.

### Sample

In view of the nature of the variables to be analyzed, a sample size calculation was made based on the application of the Student *t* -test for paired data where the mean referring of null hypothesis was 0; the significant average, of two different units; the standard deviation of 4; and the desired power, 80%, resulting in a total sample of 34 patients.

The inclusion criteria were both genders adult patients with snoring and mild, moderate and severe OSA prescribed by Sleep Physician treated with DIORS OA _m_ in the first author dental office from 2011 to 2019 period, with completed medical records and protocol of 2 to 3 months of OA _m_ adjustment. The records needed to have Epworth sleepiness scale, polysomnography and cephalometry pre- and post-treatment. The exclusion criteria were incomplete dental sleep medical records and patients that not permitted used the data for research.

The success criteria established in this study regarding elimination or decreasing of AHI symptoms 17 29 were (1) successful (AHI < 5/hour); (2), partly successful (at least 50% reduction in AHI, but AHI > 5/hour; and (3) failure (persisting clinical symptoms, and/or less than 50% reduction in baseline AHI).

In Table 1 , the characteristics of 33 adults with anthropometrics and polysomnographic and cephalometric data are showed. The anthropometric data consist in 25 men and 8 women pre- and post-treatment with mean (standard deviation) of 50.53 (10.29) years old and body mass index (BMI) of 27.70 (3.27). The angle occlusal classification of these sample is 86.96% of Class I and 13.04% of Class II. The AHI variation was 11.92 (12.80). Based on OSA severity, the sample consisted in simple snoring or normal apnea (AHI < 5) in 2.17%, mild (5 ≤ AHI < 15) in 26.09%, moderate (15 ≤ AHI < 30) in 13.04%, and severe (AHI ≥ 30) in 8.7%.

**Table 1:** Mean, standard deviation, and confidence limits of the mean (95%) that characterize sample data ( *n* = 33)

Characteristics	Mean	Standard deviation	95% confidence limit		Minimum	Maximum
			Upper	Lower		
Anthropometric data						
Cervical waist	40.76	4.59	42.44	39.07	33.50	55.00
Abdominal waist	98.98	10.21	102.73	95.24	74.00	117.00
Age	50.53	10.29	54.18	46.88	29.54	70.92
Weight (kg)	83.65	14.54	87.31	79.99	58.00	115.00
BMI (kg/m ^2^ )	27.70	3.27	28.53	26.88	20.05	37.10
Polysomnographic data						
Epworth sleepiness scale	8.22	4.53	9.36	7.08	1.00	20.00
Arousal index	9.05	8.90	11.29	6.81	0.40	53.13
SaO _2_ minimum (%)	85.57	5.65	86.99	84.15	64.00	93.00
Sleep efficiency	76.83	12.38	79.95	73.72	45.60	96.00
Apnea	4.89	8.56	7.05	2.74	0.00	43.40
Hypopnea	7.02	6.78	8.73	5.32	0.00	33.72
AHI	11.92	12.80	15.14	8.70	0.20	64.18
Minimum heartbeat	54.71	7.60	56.71	52.71	44.00	78.00
Maximum heartbeat	78.06	13.20	81.53	74.59	57.00	137.00
Legs movements	3.29	7.83	5.30	1.29	0.00	36.20
Cephalometric data						
Upper pharyngeal space	17.74	4.14	18.76	16.72	9.83	27.07
Soft palate length	41.90	5.88	43.34	40.45	28.23	58.57
Posterior air space	13.85	4.20	14.88	12.82	6.05	25.61
Hyoid distance mandibular plane	22.89	7.54	24.75	21.04	10.31	41.39
Tongue length	78.84	9.27	81.12	76.56	57.46	97.30
Tongue height	29.30	5.46	30.64	27.95	14.47	44.75
Lower air space	12.79	4.65	13.94	11.65	5.14	24.69
Hyoid distance third cervical vertebrae	42.81	5.42	44.15	41.48	32.40	53.32

Abbreviations: AHI, apnea-hypopnea index; BMI, body mass index; SaO _2_ , oxygen saturation.

### Polysomnography

Each subject underwent polysomnography all-night recordings in the sleep laboratory in two phases pre- and post-treatment. The polysomnographic study used was Brain Wave II Polysomnography (Neurovirtual, Barueri – SP/Brazil) performed by physicians specially trained in sleep medicine. The AHI was defined as the number of episodes of apnea plus episodes of hypopnea per hour of sleep. OSA was defined as AHI ≥5.

### Cephalometry

Cephalometric parameters were taken for the location of obstruction sites and to clarifying specific parameters to the upper airway with the software Radio Memory Studio 3.0 Release 7.80 (Radio Memory LTDA, Belo Horizonte – MG/Brazil). Several analysis has been widely described in literature. 14 30 31 32 33 The cephalometric analysis of airway space used and covered in two phases: without and with OA _m_ pre- and post-treatment. The majority teleradiographs and cephalometric analysis (91.3%) was performed by a single evaluator of Speed X Dental Documentation Center with Instrumentarium OC 200 Xray (KaVo company, Finland). Patients were instructed to swallow and to close their mouths with maximal intercuspation and the lips in a relaxed position when they were without and with OA _m_ .

**Fig. 2:**
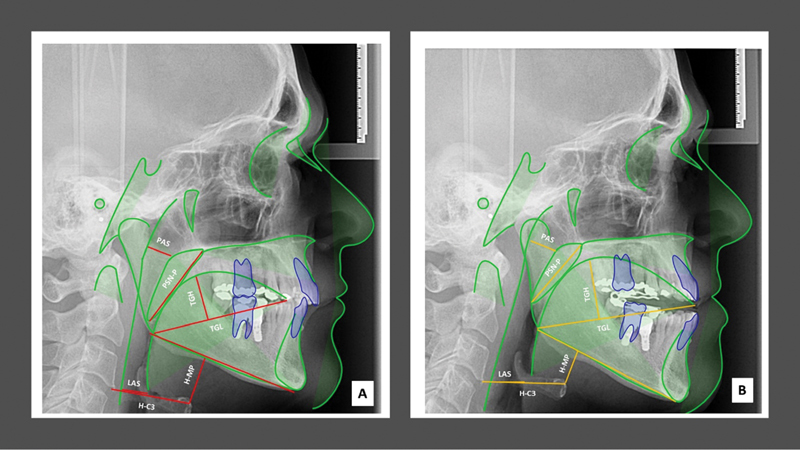
The mode of action of oral appliance with mandibular advancement used are mapped in ( **A** ) (pre-treatment) and ( **B** ) (post-treatment) in of airway space to identify tongue, soft palate, and pharyngeal airway. Lines and plans used in cephalometric variables analysis: soft palate length; posterior air space; hyoid distance third cervical vertebrae; hyoid distance mandibular plane; tongue length; tongue height; and lower air space.

Anatomically, the pharyngeal airway is divided in nasopharyngeal, oropharyngeal, and hypopharyngeal. 32 The cephalometric variables analysis of airway space used were mapped in Fig. 2A (pretreatment) and 2B (pre-treatment) showing the mode of action of OA _m_ used in the study to identify tongue, soft palate, and pharyngeal airway.

### Oral Appliance Therapy

Impressions of the dental arches and face bow to construct the gnathostatic study model based on Camper plane were made, and the constructive bite was determined by using George Gauge® bite fork.

The initial protocol of OA _m_ construction were with 65 to 75% maximum protrusion and a vertical opening of 3 to 4 mm between incisor edges. The efficacy of OA _m_ was determined by using additional PSG with OA _m_ in situ after a minimum of 3 months.

### Questionnaires

Subjective daytime sleepiness was evaluated by applying the Epworth sleepiness scale pre- and post-treatment, and a questionnaire to assess snoring, adherence, satisfaction, and safety of OA _m_ usage to partners and to the patient follow-up. The adapted questionnaire 29 consisted in following questions: Are you using OA _m_ ? If yes, mark on a scale of 1 to 3 (where: 1 = little, 2 = medium and 3 = a lot); Is your partner snoring with OA _m_ ? If yes, mark on a scale of 1 to 3 (where: 1 = little, 2 = medium and 3 = a lot); and Are you totally satisfied with OA _m_ ? Has OA _m_ already broken?

### Success Criteria

We evaluated the successful criteria of therapies with arousal index in addition to respiratory parameters, as AHI and SaO _2_ , and daytime sleepiness. Despite the lack of consensus regarding the definition of a successful criteria, 17 three successful criteria were adopted as a resolution of symptoms plus reduction of AHI: (1) success in AHI to <5/hour; (2) partial success at least 50% reduction in AHI, but AHI >5/hour; and (3) failure ongoing clinical symptoms and/or less than a 50% reduction in baseline AHI.

### Synthesis of Statistical Analysis

Descriptive analysis based in contingency tables and correlated Chi-square tests and basic statistics was used to characterize the sample. Generalized linear mixed model for repeated measure and post hoc Tukey–Kramer test compares the variables pre- and post-treatment. Residual normality was accessed by the Shapiro–Wilk Test and Pearson correlation coefficients was used to test and quantify the association between polysomnographic and cephalometric data. All analysis was calculated by using the SAS System (SAS Institute Inc. The SAS System, release 9.4. SAS Institute Inc., Cary, North Carolina, United States, 2012) and in all statistical tests the level of significance was set in 5%.

## Results

The polysomnographic data comparison pre- and post- treatment with OA _m_ ( Table 2 ) of sleep efficiency, AHI, AI, SaO _2_ , and maximum heartbeats are the greatest significance ( *p* < 0.0001). In the same way, the cephalometric data comparison regarding to the hyoid distance mandibular plane (H-MP) and upper pharyngeal space. Accompanied by other variables with significant results as the tongue length (TGL; *p* = 0.01), tongue height (TGH; *p* = 0.003), posterior air space (PAS; *p* = 0.002), and lower air space (LAS; *p* = 0.01).

**Table 2:** Cephalometric comparison of the mean (standard deviation) of the pre- and post-treatment variables with the mandibular oral appliance ( *n* = 33)

Characteristics	Phase		*p* -Value
	Pre-treatment	Post-treatment	
Anthropometric data			
BMI (kg/m ^2^ )	27.63 (3.24)	27.77 (3.24)	0.4172
Polysomnographic data			
Epworth sleepiness scale	9.70 (4.97)	6.88 (3.68)	0.0004
Arousal index	12.90 (10.91)	5.55 (4.38)	<0.0001
SaO _2_ minute (%)	82.87 (6.24)	88.03 (3.67)	<0.0001
Sleep efficiency	76.09 (11.92)	77.51 (12.93)	<0.0001
Apnea	8.57 (10.94)	1.56 (3.09)	0.0058
Hypopnea	9.28 (7.76)	4.97 (5.06)	0.0318
AHI	17.86 (15.45)	6.52 (6.19)	<0.0001
Minimum heartbeat	54.63 (7.20)	54.77 (8.06)	0.9655
Maximum heartbeat	81.42 (9.99)	75.13 (15.02)	0.0140
Legs movements	2.20 (5.17)	4.29 (9.61)	0.7172
Cephalometric data			
Upper pharyngeal space	15.90 (3.69)	19.58 (3.77)	<0.0001
Soft palate length	40.76 (5.83)	43.03 (5.80)	0.0206
Posterior air space	12.71 (4.21)	14.99 (3.94)	0.0024
Hyoid distance mandibular plane	26.51 (7.44)	19.28 (5.77)	<0.0001
Tongue length	77.75 (9.23)	79.93 (9.32)	0.0153
Tongue height	27.84 (5.20)	30.75 (5.41)	0.0039
Lower air space	11.99 (4.63)	13.60 (4.60)	0.0192
Hyoid distance third cervical vertebrae	42.41 (5.60)	43.22 (5.29)	0.3052
Oral appliance therapy			
OA _m_ advancement (%)	61.12 (15.44)	100.70 (31.11)	<.0001

Abbreviations: BMI, body mass index; OA _m_ , oral appliance with mandibular advancement; SaO _2_ minimum, minimum oxygen saturation.

The mean OA _m_ adjustment pre- and post-treatment was conducted for 61.12% (15.44) to 100.70% (31.11) showing an important advancement ( *p* < 0.0001) associated with the results.

**Fig. 3:**
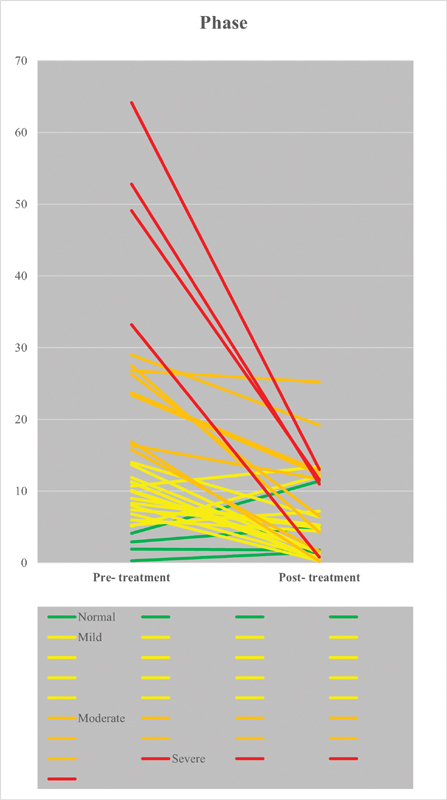
Pre- and post-treatment of obstructive sleep apnea patients without and with oral appliance with mandibular advancement.

Based on severity of AHI, the pre- and post-treatment with OA _m_ are represent in Table 3 and Fig. 3 . The frequencies and percentages in line show a significant result in different levels of AHI ( *p* = 0.0001), with 17 total success (51.51%), 9 partial success (27.27%), and 7 unsuccess (21.21%). In four patients with severe AHI, three reduce the AHI to mild and one to normal.

**Table 3:** Chi-square likelihood ratio test with frequency and percentage in line of the apnea-hypopnea index classification and treatment phase ( *n* = 33)

AHI classification	Phase	
	Pre-treatment	Post-treatment
Normal	4 (6.06)	17 (25.76)
Mild	16 (24.24)	14 (21.21)
Moderate	9 (13.64)	2 (3.03)
Severe	4 (6.06)	

Abbreviations: AHI, apnea-hypopnea index.

Note: *p* -Value = 0.0001.

The improvement in respiratory parameters was confirmed not only by comparing the polysomnographic data ( *p* < 0.0001) but also through direct associations ( Table 4 ) between AHI and both AI and maximum hazard ratio (HR), with correlation indices for AHI and AI (r = 0.87601, *p* = 0.0001) and for AHI and the maximum HR (r = 0.51025, *p* = 0.0001), respectively; and an indirect association between AHI and SaO _2_ (r = − 0.54760; *p* = 0.0008).

**Table 4:** Pearson's correlation index ( *p* -value) for quantifying the association between polysomnographic variables

	AHI		SaO _2_ (min) %		Minimum heartbeats	
SaO2 (min)%	−0.54760	(0.0001)				
Arousal index	0.87601	(0.0001)	−0.41245	(0.0008)		
Maximum heartbeats					0.51025	(0.0001)

Abbreviations: AHI, apnea and hypopnea index; SaO _2_ (min), minimum oxygen saturation.

The pharyngeal space was expanded by considering the comparison and correlation of the cephalometric measurements ( Tables 2 and 4 ). The hyoid bone presented movement in the upward and forward Table 5 , confirmed by the H-MP ( *p* < 0.0001). The greater the PAS score, the greater the LAS (r = 0.88151; *p* = 0.0001) achieved through OAT. Moreover, the correlation of the mandible-tongue relationship with the hyoid bone showed that the position of the H-MP directly correlated with the H-C3 ( *p* = 0.0034) and with the TGL ( *p* = 0.0268), pre- and post-treatment. In addition, when the mandible and tongue were protracted, there was a functional relationship between the hyoid bone and airway.

**Table 5:** Persson's correlation index ( *p* -value) to quantify the association between cephalometric variables

Variables	TGH		TGL		LAS		H-C3	
PAS	0.49702	0.0001 b	0.26976	0.0285 a	0.88151	0.0001 b	0.52311	0.0001 b
PNS-P	0.47445	0.0001 b	0.63226	0.0001 b	NS		0.38235	0.0015 b
TGH			0.65813	0.0001 b	0.30195	0.0137 b	0.43336	0.0003 b
H-MP	NS		0.27259	0.0268 a	NS		0.35549	0.0034 b
H-C3	0.43336	0.0003 b	0.55623	0.0001 b	0.47060	0.0001 b	.	

Abbreviations: H-C3, hyoid distance third cervical vertebra; H-MP, hyoid distance mandibular plane; LAS, lower air space; NS, not significant; PAS, posterior air space; PNS-P, soft palate length; TGH, tongue height; TGL, tongue length.

a *p* < 0.05.

b *p* < 0.01.

When cephalometric with polysomnographic variables were correlated, the H-MP is significantly with AHI (r = 0.44025; *p* = 0.0003) and with AI (r = 0.37683; *p* = 0.0023).

In Epworth sleepiness scale questionnaire used for evaluating subjective parameters ( Table 2 ), we observe a significant response in daytime sleepiness ( *p* = 0.0004). Furthermore, we used a questionnaire to evaluate the adherence, usage, and security of OA _m_ ( Table 6 ) showing a significant response ( *p* = 0.001) for all questions except those related to snoring. Although 12 patients continued to present snoring, it was of low intensity ( *p* = 0.0017).

**Table 6:** Frequency, percentage on the line, and Chi-square test ( *p* -value) of the answers obtained in the questionnaire applied to assess adherence, satisfaction and safety of oral appliance with mandibular advancement

Questions	Frequency	%	*p* -Value
Are you using OA _m_ ?			
No	2	6.06	<0.0001
Yes	31	93.94	
Score use of 1 to 3			
1	1	3.23	<0.0001
2	3	9.68	
3	27	87.1	
Are you using it every night?			
No	2	6.45	<0.0001
Yes	29	93.55	
Do you use OA _m_ every night in week?			
No	3	9.67	<0.0001
Yes	28	90.32	
Is your partner snoring with OA _m_ ?			
No	19	61.29	0.2087
Yes	12	38.71	
On what snoring score?			
0	18	60	0.0017
1	10	33.33	
3	2	6.67	
Are you totally satisfied with OA _m_ ?			
No	2	6.67	<0.0001
Yes	28	93.33	
Has OA _m_ ever broken?			
No	26	83.87	0.0002
Yes	5	16.13	

Abbreviation: OA _m_ , oral appliance with mandibular advancement.

Note: Score = 0 = none, 1 = little, 2 = medium and 3 = much/many.

Regarding adverse effects and symptoms from using the OA _m_ , 12 patients experienced mild and transitory adverse effects at the start of the treatment, which were resolved through massage in two patients, asymmetric adjustment in OA _m_ to adjust the mandibular posture in eight cases, and specific occlusal adjustments of imbalanced occlusion in four cases.

## Discussion

Here we provide the effectiveness of DIORS OA _m_ on the outcome of OAT by expertise of the professional and the compliance of the patient. The positive effects of OA _m_ that use Camper plane for disocclusion reference were demonstrated through comparison and correlation of objective data from polysomnography and cephalometry. In a sample of 33 patients of both genders, with primary snoring to severe OSA, we adopted the most impartial success criteria for apnea (AHI <5ev/hour) 17 resulting in 51.51% total successes to 27.27% partial success cases. Even so, the results are quite significant ( *p* < 0.0001) for AHI, AI, SaO _2_ minimum, and for the maximum heartbeats ( Table 2 ).

The OA _m_ design, construction, and individualization influence the efficacy of OAT. 3 We agree that one of the problems related to respiratory disorders is the skull structures relationship. 11 13 In addition, the dental arches must be considered with to craniofacial structures. Unfortunately, most OA _m_ are structured, using models dissociated from their relationship with craniometric structures and, consequently, without respecting the muscles involved in mandibular protrusion movements. Here, we show an OA _m_ that considers the morpho functional anatomy when seeking equilibrium for the stomatognathic system.

In polysomnographic variables, we observed the higher AHI the greater the arousal index and the greater the maximum heartbeats. In contrast, the higher the AHI, the lower the SaO _2_ . In a study of various types of OA _m_ 34 showing variable of success in the treatment of mild-to-moderate OSA to distinguish treatment responder from nonresponder patients, the authors observed that definitive device was effective in improving respiratory parameters as AHI as we observed in our data, but in the SaO _2_ , they do not observed the same result that we had observed in our findings ( *p* < 0.0001). When correlating heartbeats, both variables increase ( Table 4 ). Based on these correlations together with comparison results, the effectiveness of OAT is confirmed in the upper airway permeability improvement. In addition, contributing to the AHI and AI, it also contributed to heartbeats improvement. 8 All patients with severe OSA in this sample were treated, with three patients achieving a reduction of more than 50% in AHI and one patient reaching a normal level. In this sample, only two patients with moderate OSA did not respond to OAT. These patients presented clinically angle occlusal classification of Class I, large volume of tongue, flaccid soft palate, and cervical waist more than 40 cm. In these cases of nonresponders patients to OAT, an alternative combined treatment with positive airway pressure 10 34 or myofunctional therapy 35 and BMI control 36 can be prescribed to improve the AHI responses and patency of air space.

Based in cephalometric studies, 14 30 31 32 33 here we also demonstrate the impact of OA _m_ in upper airways with the uniquely designed OA _m_ that advances the jaw and tongue simultaneously. Our findings reinforce former studies that showed that craniofacial morphology, which included bone and soft tissues, predisposed to OSAS, reducing the permeability of the upper airways.

Anatomically, the tongue maintains several relationships with the hyoid bone 25 and, therefore, with the hyoid muscle. Several muscles of the tongue are inserted directly into the hyoid bone. Thus, the displacement of the tongue forward acts on the hyoid bone and vice versa, influencing the upward (cranial) displacement of the hyoid bone. The protrusion of the tongue or mandible, increasing the size of the airway cross-section in individuals with and without OSA, was already showed with the OAT. 14 17 37 Furthermore, the significant increase in the upper airway permeability is probable related to OA _m_ design. 18 In our findings, we demonstrate that the OA _m_ used in this study that use Camper plane as reference to disocclusion permits the jaw and the tongue simultaneous advancement and significantly increase the upper airway in OSA patients ( Fig. 2 ), confirming the effectiveness of this OA _m_ ( Fig. 3 ). This affirmative is correlating to cephalometric and polysomnographic variables, demonstrating that the position of the hyoid bone is partly a factor that can interfere with airway permeability and sleep quality contributing to the significant reduction of these indexes ( *p* < 0.0001).

In the subjective parameters, the Epworth sleepiness scale presents a significant result ( *p* < 0.0005). The questionnaire applied associated with adherence, satisfaction, symptoms, and safety of using OA _m_ ( Table 6 ), we found significant results in terms of satisfaction and adherence ( *p* = 0.0001), although there is still snoring presence, the results show an extremely low score, without disturbing the bed partner. Finally, safety of use shows that this OA _m_ is safe to use ( *p* = 0.0006).

The limitation of this study was the two-dimensional image used without provide volumetric measurements as OSA diagnostic tool, but this limitation was reduced with the follow-up polysomnography parameters. In addition, this sample was obtained in a private dental office that uses on protocol the cephalometry as a complementary diagnose tool, accessible to assist in the general assessment of soft tissues and bone configuration in the OAT. 14 30 31 32 33 Even though, the statistical data obtained are encouraging, they need to be reinforced through further investigation about OA _m_ design in larger studies to evaluate the clinical importance regard to the tongue, hyoid, mandibular posture, and the soft palate length in apneic patients pre- and post- treatment.

## Conclusion

The OAT is an important determinant in the upper airway permeability in OSA treatment, reducing snoring and daytime sleepiness. In this pilot study, DIORS OA _m_ as an uniquely designed device using Camper plane as a reference for disocclusion was effective in the control of OSA. Future studies should test and compare other OA _m_ with DIORS OA _m_ to confirm such important findings.
